# Higher‐order structure formation using refined monomer structures of lipid raft markers, Stomatin, Prohibitin, Flotillin, and HflK/C‐related proteins

**DOI:** 10.1002/2211-5463.13593

**Published:** 2023-03-17

**Authors:** Hideshi Yokoyama, Ikuo Matsui

**Affiliations:** ^1^ Faculty of Pharmaceutical Sciences Tokyo University of Science Noda Japan; ^2^ Biomedical Research Institute National Institute of Advanced Industrial Science and Technology (AIST) Tsukuba Japan

**Keywords:** *ab initio* docking, ColabFold: AlphaFold2, lipid rafts, membrane protease, SPFH, stomatin specific protease

## Abstract

Currently, information on the higher‐order structure of Stomatin, Prohibitin, Flotillin, and HflK/C (SPFH)‐domain proteins is limited. Briefly, the coordinate information (Refined PH1511.pdb) of the stomatin ortholog, PH1511 monomer, was obtained using the artificial intelligence, ColabFold: AlphaFold2. Thereafter, the 24mer homo‐oligomer structure of PH1511 was constructed using the superposing method, with HflK/C and FtsH (KCF complex) as templates. The 9mer‐12mer homo‐oligomer structures of PH1511 were also constructed using the *ab initio* docking method, with the GalaxyHomomer server for artificiality elimination. The features and functional validity of the higher‐order structures were discussed. The coordinate information (Refined PH1510.pdb) of the membrane protease PH1510 monomer, which specifically cleaves the C‐terminal hydrophobic region of PH1511, was obtained. Thereafter, the PH1510 12mer structure was constructed by superposing 12 molecules of the Refined PH1510.pdb monomer onto a 1510‐C prism‐like 12mer structure formed along the crystallographic threefold helical axis. The 12mer PH1510 (prism) structure revealed the spatial arrangement of membrane‐spanning regions between the 1510‐N and 1510‐C domains within the membrane tube complex. Based on these refined 3D homo‐oligomeric structures, the substrate recognition mechanism of the membrane protease was investigated. These refined 3D homo‐oligomer structures are provided via PDB files as Supplementary data and can be used for further reference.

## Graphical abstract

Currently, information on the higher‐order structure of lipid rafts marker, Stomatin, Prohibitin, Flotillin, and HflK/C (SPFH)‐domain proteins is limited. The homo‐24mer structure of stomatin PH1511 was constructed using AI prediction techniques. SPFH domain proteins may form huge cage structures enclosing cargo proteins. Based on these refined 3D homo‐oligomeric structures, the substrate recognition mechanism of the membrane protease was investigated.

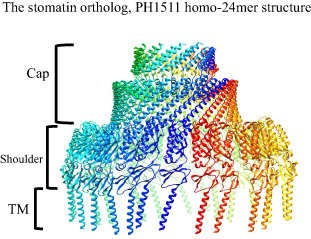

**Abbreviations**
1510‐C: residues 371–441 of PH1510
1510‐N: residues 16–236 of PH1510
KCF: HflK/C and FtsH
NfeD: nodulation formation efficiency D
OB‐fold: oligosaccharide/oligonucleotide‐binding fold
PhSto^CD^
: residues 56–234 of PH1511
CC: coiled‐coil
SPFH: Stomatin, Prohibitin, Flotillin, and HflK/C
STOPP: stomatin operon partner protein
TM: transmembrane region

Based on electron microscopy images, the lipid raft markers, Stomatin, Prohibitin, Flotillin, and HflK/C (SPFH)‐domain proteins have been proposed as circular structures comprising homo‐ or hetero‐oligomers [1, 2]. The stomatin ortholog protein (PH1511) of the hyperthermophilic archaeon, *Pyrococcus horikoshii*, was found to be poorly expressed in an *E. coli* recombinant system. Hydropathy plot analysis of the amino acid sequence revealed a single transmembrane region (residues 1–24). Further, immediately after the transmembrane region, a hydrophobic region (residues 25–54) was found to interact with the cell membrane and hydrophobic region (residues 235–245) at the C terminus. These multiple hydrophobic regions appear to lower the expression of intact molecules in *E. coli* cells. Therefore, we constructed a recombinant expression system for the soluble domain (residues 56–234, hereinafter referred to as PhSto^CD^) in *E. coli* and succeeded in protein crystallization and X‐ray crystal structure analysis. PhSto^CD^ is a novel homotrimeric structure in which three α/β domains form a 50 Å triangle with a 60 Å long α‐helical segment extending from the apex of the triangle [3]. However, this structure lacks transmembrane portions, the hydrophobic region immediately after the transmembrane region, and the hydrophobic region at the C terminus. Recently, we revealed the difference in the oligomerization state of stomatin orthologs as the core domain of mouse stomatin was reported to form a dimer [4]; however, PhSto^CD^ is a trimer. The key residues of PH1511, 56–62 and 162–168, were suggested to determine whether SPFH domains form a dimer or trimer structure [5]. As an intact 3D structure of the PH1511 monomer was lacking, it was difficult to predict a higher‐order structure consisting of homo‐oligomers.

Complexes of HflK and HflC, which are SPFH domain proteins, and FtsH, which is a membrane‐anchored AAA + protease, were purified from the *E. coli* membrane fraction after solubilization with a detergent and subjected to cryo‐electron microscopy (cryo‐EM), which revealed their high‐resolution supra‐assembly [6, 7]. The HflK‐HflC and FtsH (KCF) complex has a 2.7 MDa structure, displaying a gigantic architecture with 12 copies of the HflK‐HflC dimer to provide a circular assembly embedded in detergent molecules. Accordingly, SPFH domain proteins may form a cylindrical cage structure that protrudes from the cell membrane. The cylindrical cage structure is the main structure of lipid rafts [2].

SPFH domain proteins may form huge cage structures, which promote the functionality of membrane proteins by enclosing cargo proteins and lipids and increasing their contact frequency. However, most of their higher‐order formations and disassembly mechanisms are unknown. We constructed higher‐order structures of the stomatin ortholog, PH1511, and operon‐forming membrane protease, PH1510, using as much structural information and AI prediction techniques currently attainable, and attempted to elucidate the mechanism of disassembly for stomatin orthologs.

## Results

### PH1511 24mer structure superposed onto the HflK‐HflC and FtsH complex (KCF complex), which served as a template

Stomatin is a major integral membrane protein in erythrocytes [8]. However, stomatin is deficient in the erythrocyte membrane of patients with overhydrated hereditary stomatocytosis, a form of hemolytic anemia characterized by stomatocytic red blood cells with abnormal permeability of Na^+^ and K^+^ [8]. This deficiency may be caused by mistrafficking of the stomatin during erythropoiesis [9]. Stomatins modulate the activity of acid‐sensing ion channels [10] and GLUT‐1 glucose transporters [11]. Previously, the 3D structures of stomatin orthologs were experimentally determined, and two crystal structures have been reported. Our research group reported the first crystal structure, PhSto^CD^ [3]. The second crystal structure is the α/β core domain of the mouse stomatin [4].

As the complex structures of stomatin orthologs are unknown, we attempted to predict cylindrical cage structures using recent advances in protein structure prediction technology and artificial intelligence, and experimental data reported by us and other research groups.

**Fig. 1:**
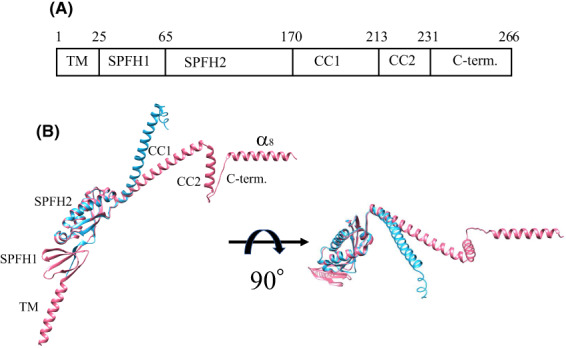
Domain organization and 3D structure of the stomatin ortholog, PH1511. (A) Domain organization of PH1511. The numbers except for the first and last ones indicate the beginning residue of each domain. Frequently used domain names are abbreviated in this fig. (B) The X‐ray crystallographic structure (PDB ID: 3BK6) in blue was superimposed onto the predicted structure (Refined PH1511.pdb) in pink. In the predicted structure (Refined PH1511.pdb), the per‐residue confidence score (pLDDT) of two short regions, (K^232^‐L^238^) and (E^263^ to end), are low (70 > pLDDT > 50). All remaining parts are very high (pLDDT > 90) or confident (90 > pLDDT > 70). The 3D structure of PH1511 was drawn using the software, chimera ver. 1.15 (UCSF, San Francisco, CA, USA).

**Fig. 2:**
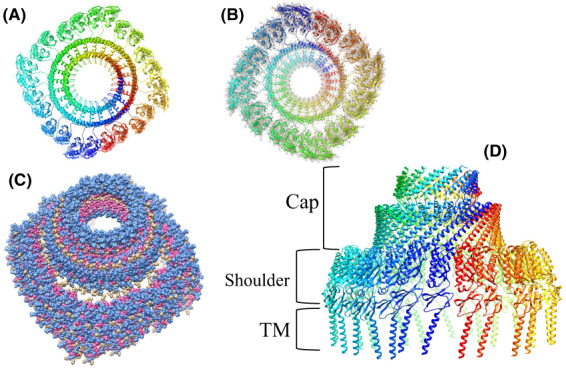
PH1511 homo‐24mer structure created using the superposing method. The structures were viewed from the top (A) and bottom (B). The figures are drawn as a ribbon model using a rainbow of colors, from purple at the 1st subunit to red at the 24th subunit. Panel (B) is also drawn with sidechains. (C) View of panel A rotated 30° around the X‐axis. The figure is shown as a space‐filling model, in which the blue and pink colors indicate polar and aliphatic amino acids, respectively. (D) Side view of (A) and (B), in which three parts, the TM, shoulder, and cap regions, are observed from the bottom to top.

Figure 1 shows the domain organization (A) and the 3D structure (B) of the PH1511 monomer based on X‐ray crystallography [3] and the structure of Refined PH1511.pdb, which was predicted by artificial intelligence, as mentioned in Materials and methods. The frequently used domain names are abbreviated in this figure. The dataset, Refined PH1511.pdb, is shown in Data S1. Figure 2 shows the PH1511 24mer structure created by superposing the Refined PH1511.pdb 24mer onto the structure of the KCF complex (7vhp.pdb) [6]. The PH1511 24mer.pdb dataset is shown in Data S2. The PH1511 24mer structure was recognized to possess a circular cavity when viewed from the C‐terminal side (Fig. 2A); however, this cavity appeared as a square when viewed from the N‐terminal side (Fig. 2B). These structural features are inherited by the shape of the KCF complex [6]. Figure 2C shows a view of Fig. 2A rotated by 30° around the X‐axis. Open spaces were observed in the regions connecting the SPFH2 and CC1 domains between the adjacent subunits. Figure 2D shows the view of Fig. 2A rotated 90° around the X‐axis. The 24mer PH1511 structure can be divided into three parts: TM, shoulder, and cap regions, where TM is a transmembrane region, the (SPFH1 + SPFH2) domain served as the shoulder region, and the (CC1 + CC2) domain plus the C‐terminal region represented the cap region. Based on the molecular structure shown in Fig. 2, the combinations that form salt bridges between subunits A and B are shown in Table 1. Accordingly, in the shoulder region, only one salt bridge was observed between the adjacent SPFH domains, and only three salt bridges were present in the (CC1 + CC2) domain. Therefore, the shoulder and cap regions may be bundled in a cylindrical shape, mainly by hydrophobic and electrostatic interactions, such as hydrogen bonds between side chains. On the contrary, the 8th helices (α_8_), which are located at the C terminus of the cap regions of the PH1511 subunits, overlapped between adjacent subunits, and several molecular clashes were observed among the side chains of α_8_. Presumably, in the native gigantic complexes, α_8_ appears to change its conformation to the outside or inside of the cylinder, and their clash states might be resolved.

**Table 1:** Salt bridges of the PH1511 cylindrical cage 24mer structure.

Region	Subunit A	Subunit B	Distance (Å)^a^
Shoulder (SPFH1 + SPFH2)	R121 HH22	D165 OD1	1.33^b^
Cap			
CC1 + CC2	R186 HH22	E194 OE1	4.40
	E194 OE2	K202 HZ2	3.03
	R204 HH11	E212 OE2	2.08
C‐term.	K249 HZ3	E256 OE2	2.98
	K260 HZ1	E264 OE1	2.36
	K261 HZ1	E265 OE1	2.66

^a^ Distances were calculated with a software chimera ver. 1.15.

^b^ This value is too small. The actual value might be much larger since these side chains would move to more appropriate position.

### Automatic prediction of a highly accurate PH1511 homomer model using the GalaxyHomomer server

**Fig. 3:**
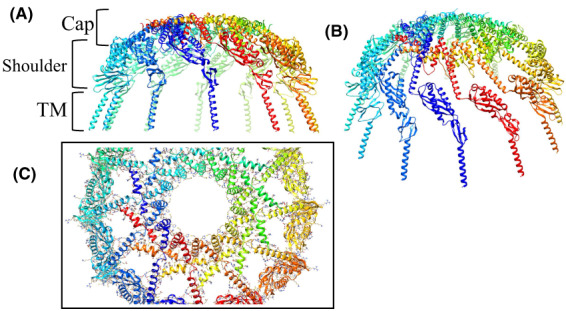
PH1511 homo‐12mer structure calculated using the *ab initio* method and the GalaxyHomomer server. The figures are drawn as a ribbon model using a rainbow of colors, from purple at the 1st subunit to red at the 12th subunit. (A) The figure is viewed from the side, in which three parts, the TM, shoulder, and cap regions, are observed from the bottom to top. (B) View of panel A rotated 30° around the X‐axis. (C) The figure is viewed from the top, in which the sidechains are also presented.

**Fig. 4:**
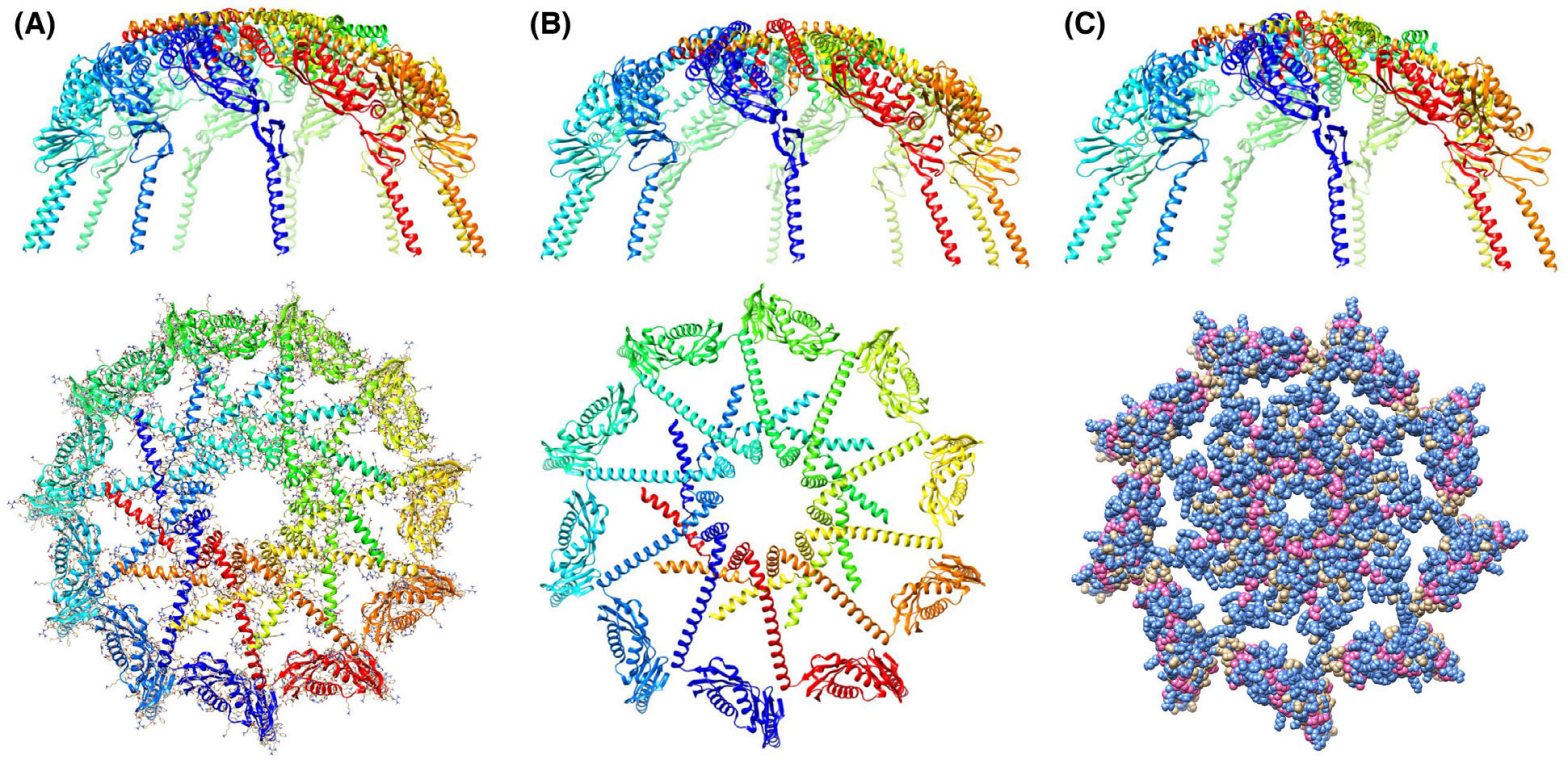
PH1511 homo‐11mer to homo‐9mer structures created using the *ab initio* method and the GalaxyHomomer server. The figures are drawn as a ribbon model using a rainbow of colors, from purple at the 1st subunit to red at the last subunit. Panels (A), (B), and (C) are homo‐11mer, homo‐10mer, and homo‐9mer structures, respectively. The upper panels are the side views. The lower panels are the top views of the upper panels. In the lower panels, the first one was drawn with sidechains, and the last one was expressed as a space‐filling model, in which the blue and pink colors indicate polar and aliphatic amino acids, respectively.

**Fig. 5:**
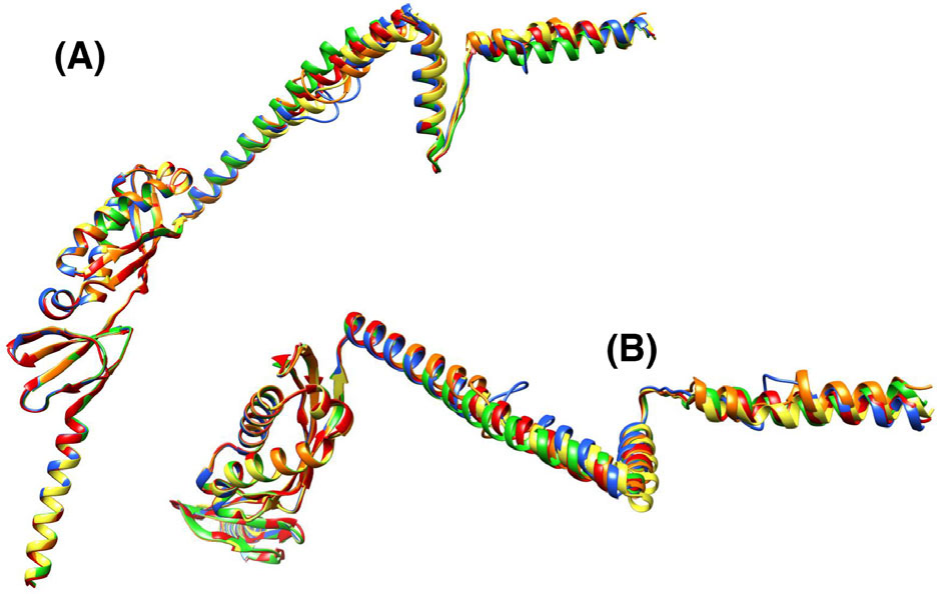
Structural comparison of the monomer in each oligomeric state superposed on the structure of Refined PH1511.pdb. (A) Monomer structures of Refined PH1511.pdb, PH1511 GH 12mer, PH1511 GH 11mer, PH1511 GH 10mer, and PH1511 GH 9mer are red, orange, yellow, green, and blue, respectively. (B) View of panel A rotated 90° around the X‐axis.

Methods for predicting the protein homo‐oligomer structure can be divided into two categories: methods that use templates selected from the protein structure database and those that dock monomer structure *ab initio*, without the use of template information. Docking methods may be more useful when proper oligomer templates are not available but the monomer structure is reliable [12]. Several protein–protein docking methods have been reported to date [13], some of which are available as public web servers for predicting homo‐oligomer structures. The GalaxyHomomer predicts the homo‐oligomer structure from either the amino acid sequence or the monomer structure [12]. The oligomeric state may or may not be specified by the user. The server can perform both template‐based oligomer modeling and *ab initio* docking and returns five model structures or automatically decides how many models are generated, depending on the existence of proper oligomer templates. The dataset, Refined PH1511.pdb, is shown in Data S1. As the template structure for the PH1511 homo‐oligomer structure has not been reported and the Refined PH1511.pdb dataset was highly refined and judged to be reliable as a monomer structure, we selected the *ab initio* docking method in the GalaxyHomomer server. As 12mer is the upper limit of the server's calculation capacity, the oligomeric state was specified from 7mer to 12mer in each calculation cycle. Unfortunately, the predicted structures of the 7mer and 8mer were not rational cylindrical cage shapes protruding from the cell membrane via transmembrane regions. Accordingly, their results were omitted from Figs 3 and 4. Thus, the predicted structures from the 12mer to 9mer of PH1511 are shown in Figs 3 and 4. The *Ab initio* docking results for the structures shown in Figs 3 and 4 are summarized in Table 2. The structures ranked as no. 1 in the same oligomeric state in Table 2 correspond to the individual structures in the same oligomeric state in Figs 3 and 4. The number 1 candidates in Table 2 have the widest interface area (Å^2^) and the highest docking score of over 800 relative to the lower ranks. This highly accurate coordinate information is called PH1511 GH 12mer. pdb, PH1511 GH 11mer.pdb, PH1511 GH 10mer.pdb, and PH1511 GH 9mer. pdb, respectively, and these datasets are shown in Data S3–S6. Based on the PH1511 GH 12mer. pdb structures shown in Fig. 3A–C, the combinations of amino acid residues forming hydrophobic interactions and salt bridges among the A–D and J–L subunits are shown in Table 3. Based on these results, no salt bridges or hydrophobic interactions exist between the adjacent SPFH domains in the shoulder region. In contrast, in the cap region, six salt bridges and 26 hydrophobic interactions were observed between the A and B–D subunits and between the A and J–L subunits. Therefore, the shoulder region has poor interaction between subunits and a high degree of freedom. On the contrary, the cap region is formed with abundant hydrophobic interactions and several salt bridges among the subunits, which could markedly contribute to homomer stability. The 3D structures of the monomer in each oligomeric state were superimposed on Refined PH1511.pdb, as shown in Fig. 5A,B, suggesting that slight structural deformations among the CC1 and CC2 domains plus the C‐term. regions were sufficient to form rational cylindrical structures variable from 9mer to 12mer. The 11mer to 9mer oligomeric states shown in Fig. 4A–C have almost the same topology as the 12mer oligomeric state shown in Fig. 3, and the cap regions mainly contribute to their homomer stabilities, despite appropriate changes to the combination of side chains contributing to the hydrophobic interactions and the salt bridges.

**Table 2:** *Ab initio* docking results for the PH1511 homomer structures predicted by the GalaxyHomomer server.

Number of subunits	Ranking no.	Interface area (Å^2^)^a^	Docking score^b^
12‐mer	1	25 371.7	816.103
12‐mer	2	10 586.4	753.081
12‐mer	3	15 471.9	700.392
12‐mer	4	12 980.8	695.962
12‐mer	5	14 723.2	684.979
11‐mer	1	23 630.0	816.183
11‐mer	2	8052.7	753.093
11‐mer	3	14 696.0	700.394
11‐mer	4	13 027.7	695.961
11‐mer	5	14 769.8	684.983
10‐mer	1	18 053.7	814.339
10‐mer	2	13 880.7	747.616
10‐mer	3	11 815.1	737.832
10‐mer	4	12 513.3	718.008
10‐mer	5	10 569.6	697.097
9‐mer	1	17 923.5	814.339
9‐mer	2	16 541.1	747.616
9‐mer	3	11 696.2	737.832
9‐mer	4	12 251.6	718.008
9‐mer	5	9328.7	697.097

^a^ Interface area (in Å^2^) between one subunit and the other subunits was calculated using the Naccess program (12)

^b^ The higher is the better score (12).

**Table 3:** Salt bridges and hydrophobic interactions between subunit A and subunits B to D and J to L of PH1511 GH 12mer.

Region	Subunit A	Subunit X^a^		Distance (Å)^b^
Salt bridges Cap (CC1 + CC2 + C‐term.)	E194 OE1	J	K266 HZ1	2.98
	R186 HH12	J	E264 OE2	2.03
	R187 HE	J	E264 OE1	2.24
	E264 OE2	D	R186 HH12	2.03
	E264 OE2	D	R187 HE	3.90
	K266 HZ1	D	E194 OE1	3.12
Hydrophobic interactions Cap (CC1 + CC2 + C‐term.)	I190 CG2	J	K266 CD	3.95
	I190 CD1	J	K266 CB	4.04
	A199 CB	K	L251 CD1	3.83
	A200 CB	K	L251 CD2	3.64
	K202 CE	K	F248 CE1	4.81
	L203 CD1	L	L240 CD2	3.98
	A206 CB	L	L217 CD2	4.54
	A207 CD1	L	L217 CD1	3.94
	L210 CG2	L	L247 CD1	3.83
	L217 CD1	L	L247 CD2	4.56
	L217 CD2	B	I209 CD1	3.78
	V237 CG1	L	L251 CD2	4.29
	L238 CD2	B	L203 CD1	4.97
	L240 CD2	B	L203 CD1	3.98
	P241 CB	L	A255 CB	4.73
	M242 CE	L	Y258 CD1	4.17
	L245 CD1	L	M259 CE	3.64
	F248 CE1	C	K202 CG	3.69
	L251 CD2	B	V237 CG1	4.29
	M259 SD	B	M239 CG	4.19
	M259 CE	B	L245 CD1	3.64
	A255 CB	C	A200 CB	4.46
	A257 CB	B	M242 CE	3.91
	Y258 CD1	B	M242 CE	4.17
	K266 CD	D	I190 CG2	3.95
	K266 CB	D	I190 CD1	4.04

^a^ Subunit X corresponds to subunits B–D and subunits J–L, respectively

^b^ Distances were calculated with the software chimera ver. 1.15.

### Fourteen‐mer (cylinder) and 12mer (prism) structures of the membrane protease, PH1510, superposed onto the cylindrical and prism‐like structures of 1510‐C according to crystal packing data

In archaeal and bacterial species, prokaryotic stomatin and stomatin operon partner protein [STOPP, also known as nodulation formation efficiency D (NfeD)] genes are thought to form an operon [14]. More than 350 archaeal and bacterial genomes have confirmed the presence of this operon [15]. The operon structure and ability of PH1510 to specifically cleave the C‐terminal hydrophobic region of the stomatin ortholog PH1511 between Leu^238^ and Met^239^ suggest that the membrane protease, PH1510, might function in cooperation with stomatin PH1511 [16].

**Fig. 6:**
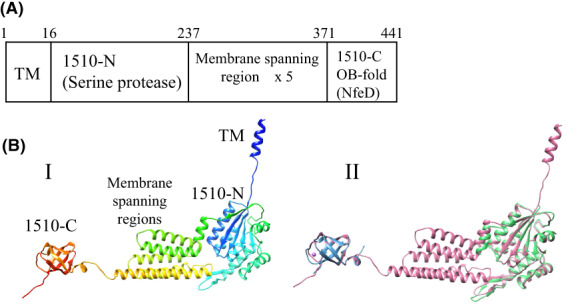
Domain organization and 3D structure of the membrane protease, PH1510. (A) Domain organization of PH1510. The numbers except for the first and last ones indicate the beginning residue of each domain. The frequently used domain names are abbreviated in this fig. (B‐I) 3D structure of PH1510 is shown as a ribbon model using a rainbow of colors, from purple at the N terminus to red at the C terminus. (B‐II) The two X‐ray structures (PDB ID: 3BPP) in green and (PDB ID: 3WWV) in blue were superimposed onto the predicted structure (Refined PH1510.pdb) in pink. In the predicted structure (Refined PH1510.pdb), the per‐residue confidence score (pLDDT) of five short regions, (M^1^‐A^20^), (Y^126^‐G^130^), (G^327^‐K^336^), (K^369^‐E^378^), and (R^435^ to end), are low (70 > pLDDT > 50). All remaining parts are very high (pLDDT > 90) or confident (90 > pLDDT > 70).

Figure 6 shows the domain organization (A) and 3D structure (B) of the membrane protease, PH1510, based on X‐ray crystallography [17, 18], and the structure of Refined PH1510.pdb, which was predicted by artificial intelligence as mentioned in Materials and methods. Frequently used domain names are abbreviated based on this figure. The dataset, Refined PH1510.pdb, is shown in Data S7. The membrane protease, PH1510 (441 residues), contained five putative membrane‐spanning regions between the N‐terminal serine protease domain (residues 16–236, 1510‐N) and the C‐terminal OB‐fold domain, alternatively termed the NfeD domain (residues 371–441, 1510‐C) as shown in Fig. 6A. The intact molecule was scarcely expressed in an *E. coli* recombinant system, presumably due to these membrane‐spanning regions. Therefore, we constructed an *E. coli* high‐expression system of the soluble N‐terminal domain (1510‐N) and successfully performed overexpression, protein crystallization and X‐ray crystal structure analysis [17]. Subsequently, we succeeded in the overexpression, crystallization, and crystal structure analysis of the soluble C‐terminal domain (1510‐C) [18]. Based on the crystal structure of 1510‐N, the 1510‐N dimer binds to one substrate peptide of stomatin PH1511 [19]. The 1510‐N dimer also exhibited protease activity, based on activity staining using casein‐copolymerized PAGE [16]. These findings indicate that the PH1510‐N dimer is a functional unit.

The crystal structure of 1510‐C has a compact five‐stranded β‐barrel fold characteristic of an OB‐fold [18]. According to the crystal packing, the 1510‐C domain can assemble into helical multimers based on a dimer as a basic unit. The 1510‐C domain also forms a large cylindrical structure composed of 28 subunits or a large triangular prism‐like structure composed of 12 subunits [18]. Our previous blue native (BN)‐PAGE results revealed that the 1510‐C domain (monomeric Mw = 9.2 kDa) produced a broadband of 110–220 kDa, indicating that the 1510‐C domain forms a 12‐ to 24‐mer homo‐oligomer [20], which is well‐matched to the multimeric structure determined by X‐ray crystallography [18].

**Fig. 7:**
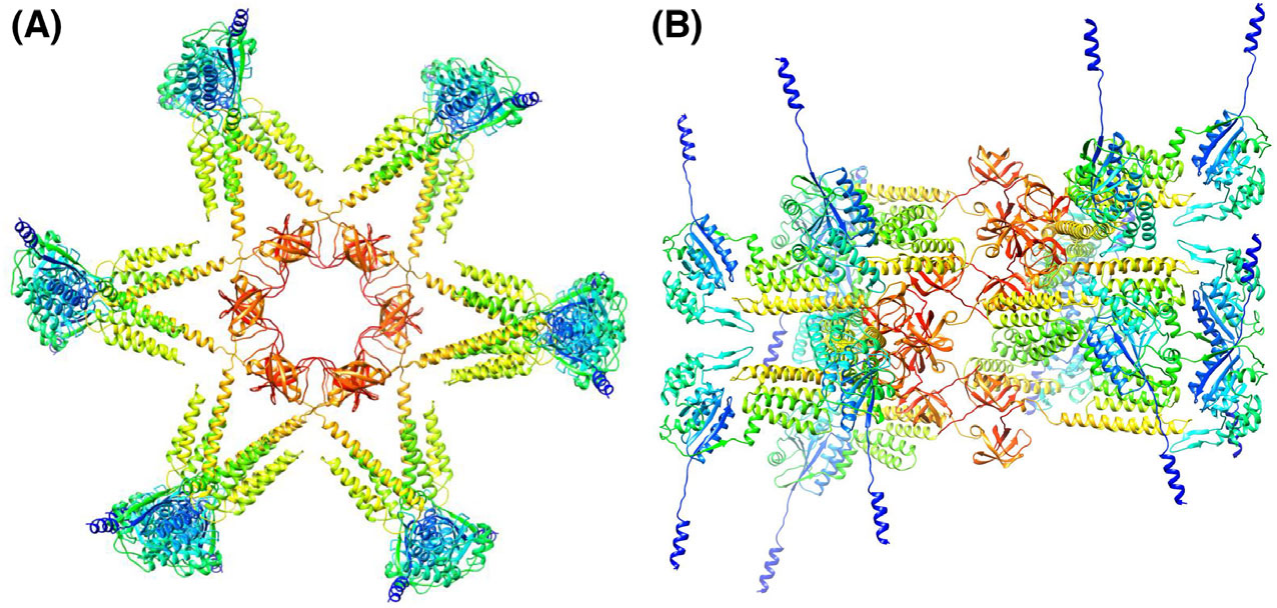
PH1510 14mer (cylinder) structure created by superposing 14 molecules of the Refined PH1510.pdb monomer onto one‐half of the cylinder‐like 28mer 1510‐C structure, a single helical structure. The structures were viewed from the top (A) and side (B). The figures are drawn as a ribbon model using a rainbow of colors, from purple at the N terminus to red at the C terminus.

We attempted to construct the PH1510 28mer structure by superposing 28 molecules of the Refined PH1510.pdb monomer onto the cylinder‐like 28mer 1510‐C structure. However, in the PH1510 28mer structure, which has a double helical structure, numerous molecular clashes were observed between several side chains, and these clashes were avoidable when the complexity of the double helical structure was eliminated to a single helical structure. Figure 7A,B show the PH1510 14mer (cylinder) structure, a single helical structure that is half the cylinder‐like 28mer structure. No molecular clash was observed in this half‐structure. The coordinate data are called PH1510 14mer (cylinder).pdb, as shown in Data S8. Figure 7B shows a view of Fig. 7A rotated by 90° around the X‐axis. As shown in Fig. 7A, the vertical section view of the cylindrical structure has an exquisite star shape.

**Fig. 8:**
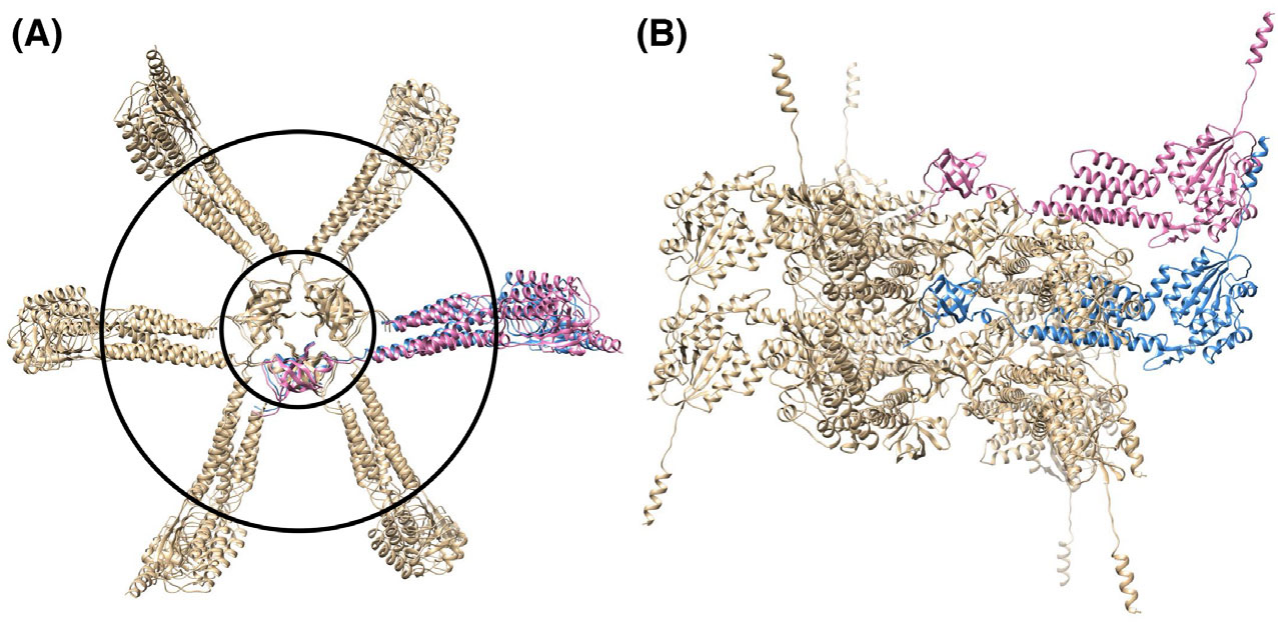
PH1510 12mer (prism) structure created by superposing 12 molecules of the Refined PH1510.pdb monomer onto the prism‐like 12mer structure of 1510‐C. The structures were viewed from the top (A) and side (B). In panel A, the outer and inner black circular lines indicate the cytosolic membrane surface and extracellular membrane surface, respectively. In panel B, two 1510‐N domains colored pink and blue might form an active serine protease dimer.

A PH1510 12mer (prism) structure was constructed by superposing 12 molecules of Refined PH1510.pdb monomers onto the prism‐like 12mer structure of 1510‐C. No molecular clash was observed in the PH1510 12mer (prism) structure, as shown in Fig. 8A,B. The coordinate data are called PH1510 12mer (prism).pdb, as shown in Data S9. As depicted in Fig. 8A, the vertical section view of the prism structure is an exquisite hexagonal (star‐shaped structure). Figure 8B shows a view of Fig. 8A rotated 90° around the X‐axis. In this figure, two 1510‐N domains colored pink and blue are located at the upper and lower positions, respectively. These two 1510‐N domains may form an active serine protease dimer [19].

## Discussion

SPFH proteins are found in all organisms, revealing an ancient protein family [21, 22, 23]. SPFH proteins usually share a tripartite domain core structure with an N‐terminal transmembrane domain, a central characteristic SPFH domain, and variable heptad repeat‐rich sequences that are predicted to form inter‐ and/or intramolecular coiled‐coil structures, termed as the CC‐domain [24, 25]. According to the fully sequenced genomes of 497 species encompassing all bacterial phyla, as well as archaea, 12 subfamilies of SPFH proteins were proposed [24]. Three subfamilies (prokaryotic stomatins, prokaryotic flotillins, and SPFH5) exhibited a conserved operon structure with STOPP. Two additional subfamilies (HflK/C proteins and prokaryotic prohibitins) are linked to the former three through functional aspects (i.e., interaction with FtsH protease). The conserved operon structure and functional similarities suggest that at least five subfamilies encompassing almost 75% of all prokaryotic SPFH members share a common origin. Their similarity to eukaryotic SPFH families and their functional similarities suggest that eukaryotic SPFH families originated from individual prokaryotic SPFH families, which may have been transferred to proto‐eukaryotes via horizontal gene transfer [24]. Thus, the coevolution scenario between the SPFH domain proteins and STOPP clearly indicates the significance of operon structures in prokaryotes [25].

Lipid rafts play essential roles in cell membrane‐mediated biological activities, such as signal transduction, active transport, and endocytosis and exocytosis [26]. Lipid rafts are also involved in the regulation of membrane fluidity by altering the membrane lipid composition. The SPFH domain proteins are the major proteins that form lipid rafts [2, 27].

The full‐length stomatin ortholog PH1511 (266 amino acid residues plus His‐tag), including hydrophobic regions (residues 1–24, residues 25–54, and residues 235–245), was weakly expressed on the *E. coli* cell membrane [20]. After solubilization with detergent LDAO, the recombinant protein was purified using a His‐tag. Using purified molecules, chemical linking with the reagent, disuccinimidyl suberate (DSS), was carried out and the resultant samples were analyzed via SDS/PAGE [20]. After 5 and 60 min of reaction, highly linked products larger than 300 kDa (molecular mass) were rapidly produced as major products and were present at the interface between the concentration gel and the separation gel of SDS/PAGE. To detect minor oligomeric products, chemically linked samples were analyzed by western blotting with an antisynthetic peptide (residues 239–253) antibody. After 5 min of reaction, 1‐, 2‐, 3‐, 6‐, 9‐, and 12‐mer bands were observed, whereas 1‐, 2‐, and 6‐mer bands were detected in the control reaction without linking. These findings indicate that the stomatin ortholog, PH1511, tends to form high‐order homo‐oligomers in detergent micelles [20].

As the PH1511 24mer structure was predicted using the KCF complex as a template, as shown in Fig. 2, structure prediction is difficult using the superposing method with fewer subunits than the 24mer owing to the lack of template structure reported. On the contrary, in the *ab initio* docking method using the GalaxyHomomer server, the number of subunits is limited to 12mer or less owing to the limited computing power of the server. As mentioned previously, a large difference exists between the 24mer structure obtained using the superposing method (Fig. 2) and the 12mer structure obtained using the *ab initio* docking method (Fig. 3). The PH1511 GH 12mer.pdb structure might be highly stable and a rational structure, because the ab initio docking structure also incorporated model refinement methods that consistently improve model quality [12]. In expectation of the next step toward the further development of the structure prediction method, if conformational changes between domains be taken into account in the ab initio docking method, the overall shapes of predicted structure from the 12mer to 9mer of PH1511 may become more similar with that of the KCF complex (7vhp.pdf). The SPFH protein cages of varying diameters and conformations could be universal modules that regulate membrane proteins involved in different cellular processes [2]. The major vault protein belonging to the SPFH domain proteins forms a cytoplasmic assembly with a 39‐fold rotational symmetry [28]. Similar to the gigantic HflK/C complex, the 39mer of the major vault protein subunit consisting of SPFH1, SPFH2, and CC domains are assembled into a cage‐like structure with a shoulder and a cap. In the cytosol, two of the 39mer vault cage further assemble to form a closed encase [28]. The assembly pattern of the CC domains for the 24mer HflK/C complex is similar to that of the 39mer major vault cage structure. The extensive atomic contacts between parallel CC1 subdomains were distributed over the entire helix and showed no bias toward polar or hydrophobic interactions. When the diameter of the stomatin ortholog PH1511 homomer is gradually increased from a 12mer to 24mer, the bias toward polar or hydrophobic interactions might be reduced, and there may be a mechanism in which the shoulder and cap regions are parallel and bundled into a cylindrical shape.

The membrane‐spanning portions of the PH1510 14mer (cylinder) structure seem to be stabilized by covering with the lipid membrane, as shown in Fig. 7. However, it is questionable whether the single helix 14mer forms a stable membrane tube complex and whether the complex could be maintained stably *in vivo*. As no molecular clash was observed and numerous membrane‐spanning portions were clustered in the PH1510 12mer (prism) structure, as shown in Fig. 8, the 1510‐C 12mer (prism) structure could be wrapped with a lipid membrane to form a stable membrane tube complex. The PH1510 12mer (prism) structure exhibits remarkably interesting characteristics. First, the triangular prism‐like structure of the 1510‐C dimer is formed along the crystallographic threefold helical axis [18]. Second, the function of the N‐terminal helix (Met^1^‐Ala^15^) is still unclear, but it might anchor the 1510‐N protease domain to the cell membrane from the cytosol. This anchoring may lead to a slight curvature of the cell membrane, resulting in membrane invagination, in which a threefold helical structure with 1510‐C dimers as the basic unit could be formed. Third, each subunit had five membrane‐spanning helices, presumably spanning the cell membrane five times. In this topology, the 1510‐N protease domain is located at the cytosol side, and the 1510‐C domain is inevitably present on the extracellular side, suggesting that membrane pits are formed by invagination from the extracellular side. As shown in Fig. 8B, two 1510‐N domains, colored pink and blue, are located at the upper and lower positions. Focusing on the locations of the two 1510‐N domains, these two 1510‐N domains always exist at the tips of the clustered membrane‐spanning helices extended in the same direction from the same edge of the prism structure, as shown in Fig. 8A, which serves as the fourth interesting property. With such structural regularity, the active 1510‐N dimer is formed along the crystallographic threefold helical axis.

As mentioned previously, the 12mer (prism) molecular structure revealed the relative positions of membrane‐spanning regions between the 1510‐N and 1510‐C domains within the membrane tube (Figs. 8A,B). The hexagonal distribution of the 1510‐N protease dimer might be suitable for multiple attacks on hydrophobic clusters consisting of C‐terminal hydrophobic regions (V^235^‐I‐V‐L‐M‐L‐P‐M^242^) of the stomatin PH1511 homomer [16]. Five α‐helices that constitute the membrane‐spanning region of each subunit form a bundle structure in the cell membrane. In the first step, the assembly of a pair of bundles aligned vertically might occur via hydrophobic interactions in the cell membrane, followed by rotation of the apical 1510‐N domains, leading to the formation of the active dimer‐type structure previously reported by our group [19]. As shown in Fig. 8B, two 1510‐N domains colored pink and blue were aligned vertically at the upper and lower positions. To form the active 1510‐N dimer, the subunit colored pink rotates 90° clockwise around the X‐axis, and the subunit colored blue rotates 90° counterclockwise around the X‐axis. Consequently, the two active center Ser^97^‐Lys^138^ dyads face each other [19].

In human stomatin, a small C‐terminal region consisting of largely hydrophobic residues, Ser^264^‐Thr‐Ile‐Val‐Phe‐Pro‐Leu‐Pro‐Ile^272^ (corresponding to residues 234–242 of PH1511), was demonstrated to be crucial for oligomerization [29]. The introduction of Ala substitutions in region 264–272 resulted in the appearance of a monomer, which was detected via SDS/PAGE. As the diameter of the hexagonal section of the PH1510 12mer (prism) structure was almost the same as the diameter of the outer boundary of the shoulder region of the PH1511 24mer, the PH1510 12mer (prism) membrane complex could not be housed in a PH1511 24mer cylindrical cage. Assuming a stage in which the PH1511 24mer is separated into a few oligomeric clusters, the activated 1510‐N protease dimer could be accessible to the C‐terminal hydrophobic region (V^235^‐I‐V‐L‐M‐L‐P‐M^242^) of the homo‐oligomer PH1511. The same situation was observed between the activated 1510‐N protease dimer and the PH1511 GH oligomeric clusters.

In conclusion, the study findings confirmed the significant roles of the (CC1 + CC2) domains in the formation of cylindrical cages of SPFH domain proteins as the main bodies of lipid rafts. The importance of no bias toward polar or hydrophobic interactions in the (CC1 + CC2) domains between subunits was also suggested to maintain the dynamic equilibrium of the formation and disassembly of the cage structure. Furthermore, the stabilities of the 1510‐C domain cores with prism structures around the threefold helical axes in the membrane tube complexes are important for the efficient attack of the 1510‐N protease on the C‐terminal hydrophobic sequences essential for the assembly of the SPFH domain proteins. SPFH protein cages of varying diameters and conformations could be universal modules that regulate membrane proteins involved in cellular processes that are important for several human diseases [2]. Stomatin and its partner protease offer a scientific basis for studying the dynamics and regulation of SPFH cage assembly and disassembly, which could influence membrane morphology and fluidity, thereby providing new insights into this elusive but significant subject.

## Materials and methods

### ColabFold: AlphaFold2 using MMseqs2

Recent advances in protein structure prediction using artificial intelligence have been remarkable; AlphaFold2 is a representative example [30]. The high accuracy of AlphaFold2 for many predicted structures was demonstrated in the latest round of protein folding competition by the international community, CASP14 (Critical Assessment of Protein Structure Prediction, round 14) [31]. In this study, ColabFold: AlphaFold2 [30] was used to obtain the coordinate information (Refined PH1511.pdb) described in the PDB file format of the highly refined three‐dimensional (3D) structure of the stomatin ortholog, PH1511 monomer. Similarly, coordinate information (Refined PH1510.pdb) of the highly refined 3D structure of the specific membrane protease PH1510 monomer was obtained. PH1510 forms an operon with PH1511 and hydrolyzes the C‐terminal hydrophobic region (V^235^‐I‐V‐L‐M‐L‐P‐M^242^) of PH1511 as a substrate [16].

We accessed the ColabFold server using the following URL: https://colab.research.google.com/github/sokrypton/ColabFold/blob/main/AlphaFold2.ipynb.

The UniProtKB Entry numbers for the amino acid sequences of the stomatin ortholog, PH1511, and the membrane‐associated protease, PH1510, are O59180 and O59179, respectively.

None template mode and use_amber mode were selected. MMseqs2 (UniRef+Environmental) and unpaired+paired were selected for msa_mode and pair_mode, respectively. Model_type and num_recycles are auto and three, respectively. As five models are proposed automatically by the ColabFold: AlphaFold2 server, a dataset (PH1511_16763_relaxed_rank_1_model_4. pdb) and another dataset (PH1510_24754_relaxed_rank_1_model_3. pdb) were selected as the Refined PH1511.pdb and Refined PH1510.pdb datasets, respectively.

### PH1511 24mer molecular structure prediction using the KCF structure (7vhp.pdb) as template

A PH1511 24mer molecular structure was constructed by superposing 24 molecules of Refined PH1511.pdb monomers on the KCF complex as a template, which consisted of 12 copies of HflK‐HflC dimers, using the SSM superpose function of the protein modeling software, COOT. First, the Refined PH1511.pdb monomer was superimposed onto one monomer of HflK‐HFlC. As a result, the domains of TM, SPFH1, and SFPH2 were well superimposed. Second, the cap region domains (CC1, CC2, and C‐term.) of the Refined PH1511.pdb monomer were superimposed onto the corresponding domains of one monomer of HflK‐HflC. As the CC1 domain of PH1511 is shorter than that of the KCF complex, the superposed TM and shoulder (SPFH1 and SPFH2) regions of the PH1511 24mer were located far away from the superposed cap region (CC1, CC2, and C‐term.) of the PH1511 24mer. As Ala^170^ is the residue located at the boundary of the SPFH2 and CC1 domains of the PH1511 24mer, the cap region of the PH1511 24mer was connected to the shoulder region of the PH1511 24mer using Ala^170^ as the region boundary.

### Modeling the PH1511 homo‐oligomer structures from 7mer to 12mer with GalaxyHomomer: A web server for protein homo‐oligomer structure prediction from a monomer structure

Homo‐oligomerized proteins are abundant in nature and are often closely related to their physiological functions. Information on homo‐oligomer structure is therefore important for understanding protein functions at the molecular level. The PH1511 homo‐oligomer structure was predicted as described below. Based on the monomer structure (Refined PH1511.pdb) predicted by ColabFold: AlphaFold2 using MMseqs2 [30], we used the Galaxy Homomer server [12] to rationally predict cylindrical cage structures by *ab initio* docking. The GalaxyHomomer server is freely accessible at http://galaxy.seoklab.org/homomer, and serial homo‐oligomer structures of PH1511 from 7mer to 12mer were calculated.

### Rational modeling of the 28mer and 12mer PH1510 structures based on X‐ray structural data for the C‐terminal soluble domain of PH1510 (1510‐C)

According to crystal packing, the 1510‐C domains are assembled into helical multimers based on a dimer as a basic unit. The 1510‐C domain forms a large cylindrical structure composed of 28 subunits or a large triangular prism‐like structure composed of 12 subunits [18]. The 28mer and 12mer PH1510 structures were constructed by superposing the Refined PH1510.pdb structure onto the 28‐mer cylinder‐like 1510‐C structure and 12‐mer prism‐like 1510‐C structure, respectively. The SSM superposing function of the model building software, COOT, was used for modeling by referring to the following site to obtain and set up the program COOT: http://www.ysbl.york.ac.uk/emsley/coot/.

## Conflict of interest

The authors declare no conflict of interest.

### Peer review

The peer review history for this article is available at https://www.webofscience.com/api/gateway/wos/peer‐review/10.1002/2211‐5463.13593.

## Author contributions

HY and IM conceived and designed the project; HY and IM acquired the data; HY and IM analyzed and interpreted the data; IM wrote the paper.

## Supporting information


**Data S1.** Refined PH1511.pdb.

**Data S2.** PH1511 24mer.pdb.

**Data S3.** PH1511 GH 12mer.pdb.

**Data S4.** PH1511 GH 11mer.pdb.

**Data S5.** PH1511 GH 10mer.pdb.

**Data S6.** PH1511 GH 9mer.pdb.

**Data S7.** Refined PH1510.pdb.

**Data S8.** PH1510 14mer (cylinder).pdb.

**Data S9.** PH1510 12mer (prism).pdb.

## Data Availability

The supporting data were contained in the manuscript as Supplementary Information.
